# Ischemic Postconditioning Alleviates Intestinal Ischemia-Reperfusion Injury by Enhancing Autophagy and Suppressing Oxidative Stress through the Akt/GSK-3*β*/Nrf2 Pathway in Mice

**DOI:** 10.1155/2020/6954764

**Published:** 2020-03-14

**Authors:** Rong Chen, Yun-yan Zhang, Jia-nan Lan, Hui-min Liu, Wei Li, Yang Wu, Yan Leng, Ling-hua Tang, Jia-bao Hou, Qian Sun, Tao Sun, Zi Zeng, Zhong-yuan Xia, Qing-tao Meng

**Affiliations:** ^1^Department of Anesthesiology, Renmin Hospital of Wuhan University, Wuhan, Hubei 430060, China; ^2^Zhongnan Hospital of Wuhan University, Hepatobiliary Diseases of Wuhan University, Transplant Center of Wuhan University, Hubei Key Laboratory of Medical Technology on Transplantation, Wuhan, Hubei 430071, China

## Abstract

**Aims:**

Ischemic postconditioning (IPO) has a strong protective effect against intestinal ischemia-reperfusion (IIR) injury that is partly related to autophagy. However, the precise mechanisms involved are unknown.

**Methods:**

C57BL/6J mice were subjected to unilateral IIR with or without IPO. After 45 min ischemia and 120 min reperfusion, intestinal tissues and blood were collected for examination. HE staining and Chiu's score were used to evaluate pathologic injury. We test markers of intestinal barrier function and oxidative stress. Finally, we used WB to detect the expression of key proteins of autophagy and the Akt/GSK-3*β*/Nrf2 pathway.

**Results:**

IPO significantly attenuated IIR injury. Expression levels of LC3 II/I, Beclin-1, and p62 were altered during IIR, indicating that IPO enhanced autophagy. IPO also activated Akt, inhibited GSK-3*β*/Nrf2 pathway.

**Conclusion:**

Our study indicates that IPO can ameliorate IIR injury by evoking autophagy, activating Akt, inactivating GSK-3*β*, and activating Nrf2. These findings may provide novel insights for the alleviation of IIR injury.*β*/Nrf2 pathway.

## 1. Introduction

Ischemia-reperfusion (IR) injury is a phenomenon in which the reperfusion of ischemic organs or tissues aggravates their damage [1]. Intestinal ischemia-reperfusion (IIR) is observed in many clinical situations, such as intestinal obstruction, strangulation, mesenteric artery thrombosis, trauma, shock, and intestinal transplantation [2]. As one of the most sensitive organs to IR injury, IIR can be a life-threatening pathological event that not only causes local tissue injury but often leads to systemic inflammatory response syndrome (SIRS) and multiple organ dysfunction syndrome (MODS) with high morbidity and mortality [3]. Previous studies have shown that oxidative stress plays an important role in IIR injury [4]. The excessive generation of reactive oxygen species (ROS) in damaged tissues and cells during IIR injury activates a variety of signaling pathways, promotes the inflammatory reaction, and damages the function of the intestinal mucosal barrier.

Ischemic postconditioning (IPO) is defined as repetitive brief periods of ischemia followed by short intervals of reperfusion before the final restoration of blood flow [5]. IPO has been shown to have a strong protective effect against IR injury in the heart, brain, liver, kidney, and spinal cord [6–8]. Nevertheless, the identification of the mechanism related to the multiple and interacting components of this process requires further study.

As a conserved cytoprotective process, autophagy degrades and recycles damaged proteins and organelles via lysosomal degradation and is indispensable for cell homeostasis under normal conditions; therefore, autophagy facilitates cytoprotective responses to adverse conditions [9]. Nevertheless, when IR-induced ROS accumulation exceeds autophagic clearance, autophagy fails to remove all dysfunctional organelles and ultimately leads to cell death [10]. Insufficient autophagy is a pivotal mechanism underlying IIR injury. Intriguingly, both IPO [11] and ischemic preconditioning [12] protect tissue from IR injury by activating autophagy. However, the contribution of the autophagic mechanism to IPO-afforded protection and the potential mechanisms involved are not understood fully.

As a redox-sensitive transcription factor, nuclear factor erythroid 2-related factor 2 (Nrf2) has a role in the pathogenesis of many diseases by coordinating the controlled expression of antioxidant genes to reinstate redox homeostasis in the presence of excessive oxidation. After exposure to oxidative stress, Nrf2 dissociates from Keap1, translocates into the nucleus, and binds to antioxidant responsive element (ARE) sequences in the promotor region of antioxidant genes [13]. Although the mechanism by which Nrf2 is liberated from the Keap1-Nrf2 complex remains to be established, recent studies have suggested that it is regulated by multiple upstream regulatory molecules. Furthermore, Nrf2 has been demonstrated to be involved in the activation of autophagy [14, 15].

Glycogen synthase kinase 3 beta (GSK-3*β*) is a constitutively activated Ser/Thr protein kinase that regulates glycogen metabolism, gene expression, and apoptosis [16]. It plays a pivotal role in mitochondria-mediated cell death. When GSK-3*β* is inhibited by Akt via phosphorylation of the serine 9 residue, it suppresses the opening of the mitochondrial permeability transition pore (mPTP) and reduces mitochondrial membrane potential (MMP), thereby reducing ROS production and contributing to the protection of mitochondria against oxidative stress [17]. In addition, several lines of evidence indicate that GSK-3*β* is a novel regulator of Nrf2 [18], suggesting that Nrf2 may function in cooperation with the Akt/GSK-3*β* signaling pathway. Therefore, in this work, we analyzed the role of autophagy in IPO-afforded protection, as well as the regulatory mechanisms of autophagy, particularly its linkage to the Akt/GSK-3*β*/Nrf2 signaling pathway.

## 2. Results

### 2.1. IPO Protects the Gut against IIR Injury

**Figure 1:**
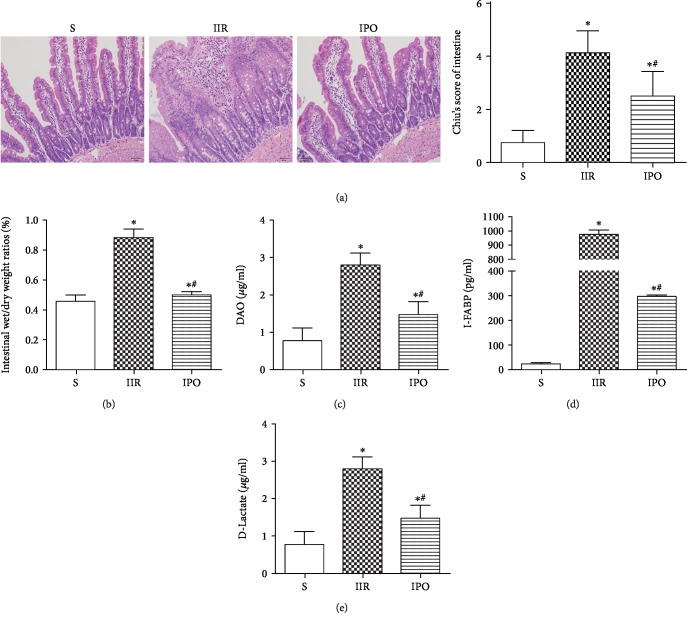
IPO alleviates IIR-induced intestinal injury in mice. (a) Histopathologic changes of the intestine under a light microscope (hematoxylin-eosin staining, ×200, scale bar 50 *μ*m). Intestinal mucosal injury was graded by Chiu's score. (b) Intestinal water content was used to assess gut permeability. Serum concentrations of DAO (c), I-FABP (d), and D-LA (e) were detected to determine intestinal epithelial function. Data are presented as the mean ± SD (*n* = 8). ^∗^*p* < 0.05 versus S; ^#^*p* < 0.05 versus IIR.

Sham (S) group mice exhibited normal morphology in terms of intestinal histology. IIR-induced intestinal injury with denuded villi, capillary congestion, and an increased gap between epithelial cells was significantly attenuated by IPO. Chiu's scores were parallel with the histological changes of the intestine (Figure 1(a), *p* < 0.05).

To characterize the lesions induced by IIR, we assessed the intestinal wet/dry (W/D) weight ratio as an indicator of the damage to gut permeability. The intestinal W/D ratio was significantly elevated in the IIR group compared with the S group (*p* < 0.05) and decreased significantly after IPO (*p* < 0.05) (Figure 1(b)). Furthermore, the serum concentrations of diamine oxidase (DAO) (Figure 1(c)), intestinal fatty acid binding protein (I-FABP) (Figure 1(d)), and D-lactate acid (D-LA) (Figure 1(e)) were used as biomarkers to estimate the function of the intestinal epithelium. The serum levels of DAO, I-FABP, and D-LA were notably increased in the IIR group compared with the S group (*p* < 0.05). As expected, the levels of these biomarkers were significantly lower in the IPO group (*p* < 0.05).

### 2.2. IPO Suppresses Oxidative Stress and Evokes Intestinal Autophagy

**Figure 2:**
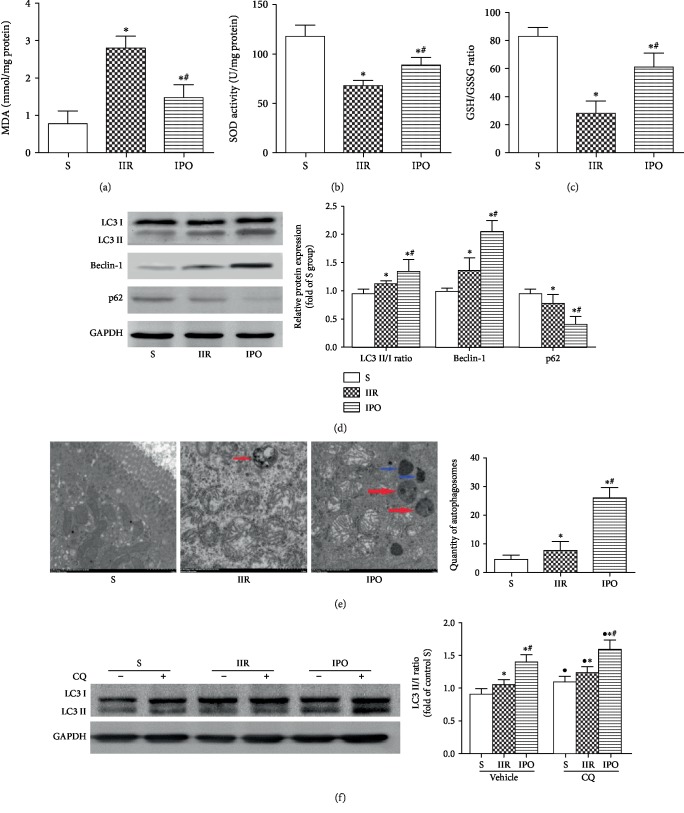
IPO suppresses oxidative stress and evokes intestinal autophagy during IIR-induced intestinal injury in mice. MDA levels (a), SOD activity (b), and the GSH/GSSG ratio (c) were determined using colorimetric assays. (d) Expression of key molecules involved in autophagy in the gut was detected by western blot analysis in each group. (e) Autophagosomes were observed under an electron microscope (red arrows point to autophagosomes, blue arrows point to autolysosomes, scale bar 1.0 *μ*m). Histogram shows the average number of autophagosome structures per view (371 *μ*m^2^) obtained by examining at least 50 images per testing sample. (f) Representative immunoblots for the LC3 II/I ratio and GAPDH with and without CQ. Data are presented as the mean ± SD (*n* = 8). ^∗^*p* < 0.05 versus S; ^#^*p* < 0.05 versus IIR.

The MDA level (Figure 2(a)) was markedly increased, and SOD activity (Figure 2(b)) and the GSH/GSSG ratio (Figure 2(c)) were significantly decreased in the IIR group compared with the S group (*p* < 0.05). IPO significantly upregulated SOD activity and the GSH/GSSG ratio and significantly downregulated the increased MDA level compared with the IIR group (*p* < 0.05). As IIR-induced oxidative stress was alleviated by IPO, we examined whether IPO could enhance autophagic clearance capacity, which would counterbalance the accumulation of ROS caused by dysfunctional mitochondria. We examined the expression of autophagy-related proteins in the intestine (Figure 2(d)). Overtly elevated levels of LC3 II/I and Beclin-1 and reduced levels of p62 were revealed following IIR insult (*p* < 0.05), and this tendency was much more pronounced with IPO (*p* < 0.05). TEM was used to analyze the ultrastructure of enterocytes. An increased number of autophagosomes were found in the IIR group, and this trend was even more marked with IPO (Figure 2(e), *p* < 0.05). As a lysosome inhibitor, Chloroquine (CQ) can block the amalgamation of autophagosomes with lysosomes to inhibit autophagic flux. CQ pretreatment before the experiments resulted in further upregulation of the LC3 II/I ratio (Figure 2(f), *p* < 0.05).

### 2.3. Defective Autophagy Abrogates the Protective Effects of IPO

**Figure 3:**
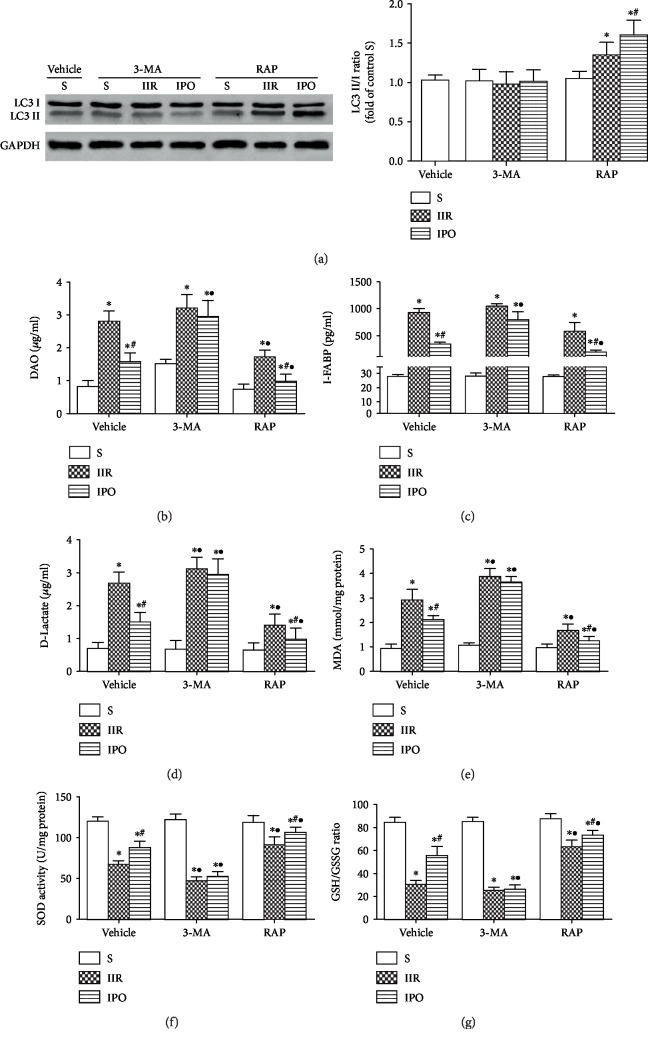
Defective autophagy abrogates the protective effects of IPO. Mice were pretreated with 3-MA or RAP prior to ischemia. (a) Autophagic protein LC3 in the ischemic gut was examined by western blot analysis. Levels of serum DAO (b), I-FABP (c), and D-LA (d) were analyzed as a measure of tissue injury. MDA levels (e), SOD activity (f), and the GSH/GSSG ratio (g) were determined using colorimetric assays. Data are presented as the mean ± SD (*n* = 8). ^∗^*p* < 0.05 versus S; ^#^*p* < 0.05 versus IIR; ^•^*p* < 0.05 versus vehicle.

To determine the role of autophagy in the protective effects of IPO, autophagy was suppressed by the established pharmacological inhibitor 3-MA or induced by rapamycin. Western blot analysis revealed that 3-MA treatment abolished the increase of the LC3 II/I ratio induced by IPO, and there was no statistical difference between the S+3-MA, IIR+3-MA, and IPO+3-MA groups (Figure 3(a), *p* > 0.05), indicating that autophagy was inhibited. Furthermore, 3-MA augmented the IIR-induced increases in serum DAO (Figure 3(b)), I-FABP (Figure 3(c)), and D-LA (Figure 3(d)) and tissue MDA levels (Figure 3(e)), while intestinal SOD activity (Figure 3(f)) and the GSH/GSSG ratio (Figure 3(g), *p* < 0.05) were decreased. Additionally, 3-MA abolished the protection conferred by IPO. Conversely, pretreatment with rapamycin increased the LC3 II/I ratio (Figure 3(a), *p* < 0.05), indicating that autophagy was activated. Rapamycin treatment decreased serum DAO (Figure 3(b)), I-FABP (Figure 3(c)), and D-LA (Figure 3(d)) and tissue MDA levels. Rapamycin also prevented the decrease in SOD activity (Figure 3(f)) and the GSH/GSSG ratio (Figure 3(g)) in the IIR group (*p* < 0.05). The combination of IPO with rapamycin made the above changes even more pronounced (*p* < 0.05).

### 2.4. Effects of IPO on Intestinal Akt, GSK-3*β*, and Nrf2 Signaling

**Figure 4:**
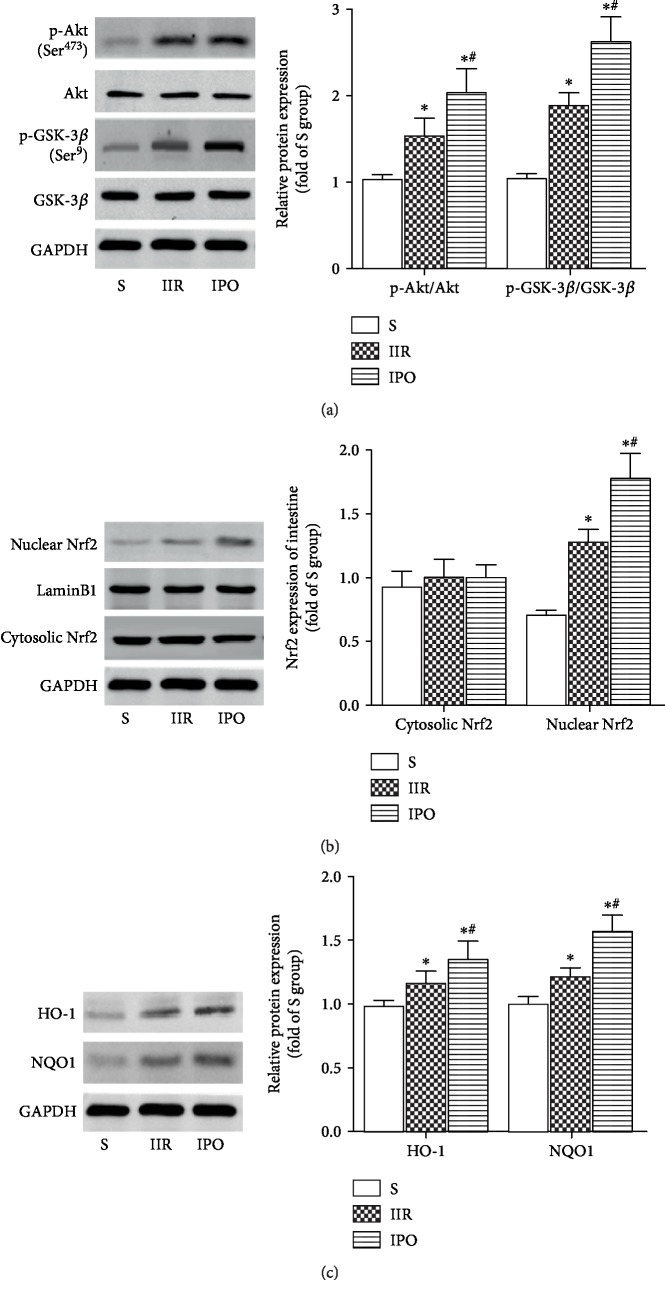
IPO activates Akt, inhibits GSK-3*β*, induces Nrf2 nuclear translocation, and upregulates HO-1 and NQO1 expression. (a) Expression of p-Akt (Ser^473^), Akt, p-GSK-3*β* (Ser^9^), and GSK-3*β*. Expression of Nrf2 (b) (in the nucleus and cytoplasm) and its downstream targets HO-1 and NQO1 (c) was detected by western blot analysis in each group. Data are presented as the mean ± SD (*n* = 8). ^∗^*p* < 0.05 versus S; ^#^*p* < 0.05 versus IIR.

IIR insult activated p-Akt and p-GSK-3*β*, which were increased further by IPO (Figure 4(a), *p* < 0.05). We then further investigated the protein expression of Nrf2 and downstream targets. Compared with the S group, the accumulation of Nrf2 protein in the nucleus was slightly increased in the IIR group and was clearly enhanced in the IPO group (Figure 4(b), *p* < 0.05). Consequently, the protein expression of two downstream targets of Nrf2, HO-1 and NQO1, was remarkably increased in the IPO group compared with the IIR group (Figure 4(c), *p* < 0.05).

### 2.5. IIR-Induced Nrf2 Activation May Be Caused by Akt-Mediated GSK-3*β* Phosphorylation

**Figure 5:**
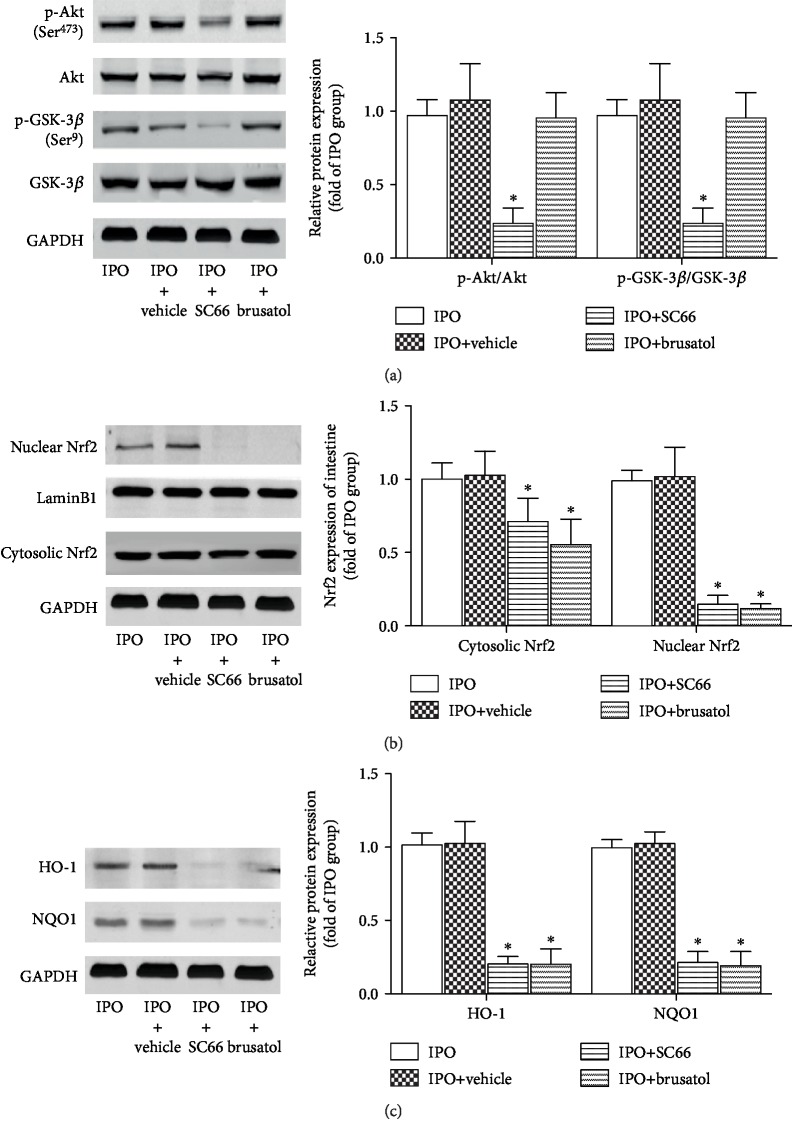
Protective effects of IPO depend on Akt/GSK-3*β*-mediated Nrf2 activation. Mice were pretreated with SC66 or brusatol prior to ischemia. (a) Expression of p-Akt (Ser^473^), Akt, p-GSK-3*β* (Ser^9^), and GSK-3*β*. Expression of Nrf2 (b) (in the nucleus and cytoplasm) and its downstream targets HO-1 and NQO1 (c) was detected by western blotting in each group. Data are presented as the mean ± SD (*n* = 8). ^∗^*p* < 0.05 versus IPO.

To identify the relationship between the Akt/GSK-3*β* and Nrf2/ARE pathways in the protection of IPO, we used the inhibitors SC66 and brusatol, respectively. SC66 treatment decreased p-Akt, p-GSK-3*β*, Nrf2, HO-1, and NQO1 in the IPO+SC66 group compared with IPO (*p* < 0.05) but did not affect total Akt and GSK-3*β* protein expression (Figures 5(a) and 5(b)). After treatment with brusatol, the expression of Nrf2, HO-1, and NQO1 was markedly downregulated (Figure 5(b), *p* < 0.05), but there was no effect on Akt and GSK-3*β* protein expression and their phosphorylation.

### 2.6. Protective Effects of IPO Depend on Akt/GSK-3*β*/Nrf2-Mediated Autophagy

**Figure 6:**
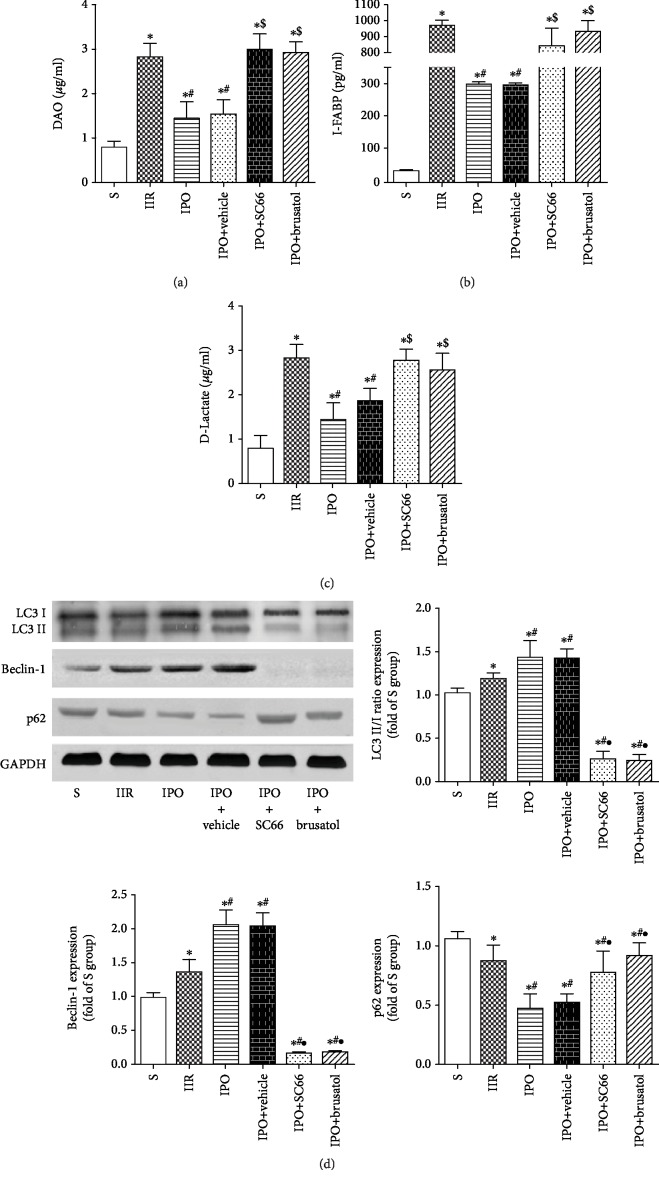
Protective effects of IPO depend on the activation of Akt/GSK-3*β*/Nrf2-mediated autophagy. Mice were pretreated with SC66 or brusatol prior to ischemia. Levels of serum DAO (a), I-FABP (b), and D-LA (c) were analyzed as a measure of tissue injury. (d) Expression of LC3, Beclin-1, and p62 was detected by western blot analysis in each group. Data are presented as the mean ± SD (*n* = 8). ^∗^*p* < 0.05 versus S; ^#^*p* < 0.05 versus IIR; ^•^*p* < 0.05 versus IPO.

Treatment with SC66 and brusatol significantly increased serum DAO (Figure 6(a)), I-FABP (Figure 6(b)), and D-LA (Figure 6(c)) levels (*p* < 0.05). Moreover, SC66 and brusatol also downregulated the LC3 II/I ratio and Beclin-1 expression, while they upregulated p62 expression (Figure 6(d), *p* < 0.05).

## 3. Discussion

We previously demonstrated that IPO protects the intestine and remote organs from IIR injury [19, 20]. In the present study, we demonstrated that IPO-induced autophagy had a crucial protective role against IIR injury. The suppression of autophagy by pretreatment with the inhibitor 3-MA contributed to a greater vulnerability to IIR insult and abolished the beneficial effects of IPO. IPO-induced autophagy was associated with activated Akt, inhibited GSK-3*β*, induced Nrf2 nuclear translocation, and upregulated HO-1 and NQO1 expression. Our study suggests that the activation of the Akt/GSK-3*β*/Nrf2 signaling pathway plays a pivotal role in IPO-mediated protection against IIR injury through the stimulation of autophagy and attenuation of oxidative stress.

In 2003, Zhao et al. [21] first proposed IPO as a potent endogenous protective mechanism to protect the myocardium from IR injury. IPO is defined as rapid intermittent interruptions of blood flow in the early phase of reperfusion that mechanically alter the hydrodynamics of reperfusion. This endogenous protective-adaptive intervention suppresses spontaneous Ca^2+^ oscillations and inhibits mPTP opening [22], contributing to the maintenance of mitochondrial homeostasis, attenuation of oxidative stress, and alleviation of apoptosis [23]. IPO-induced protection against IR injury has been confirmed in multiple organs [11, 19, 22]. Consistent with these studies, we found that IPO markedly attenuated IIR injury as characterized by reduced histological damage and lower levels of serum biomarkers. Furthermore, this protective effect was associated with ameliorated oxidative stress by decreasing lipid peroxidation and increasing antioxidant capacity. Some studies have demonstrated that the beneficial effects of IPO are associated with its ability to activate autophagy. Nevertheless, the contribution of the autophagic mechanism to IPO-afforded protection and the underlying mechanisms involved are still not understood fully.

Autophagy is an evolutionarily conserved, cellular self-defense response involving autophagosome formation and lysosomal degradation of dysfunctional cytoplasmic organelles or damaged cytosolic components [24]. The execution of autophagy involves three critical proteins, namely, LC3 II, p62, and Beclin-1. Accumulating evidence has shown that autophagy participates in the pathological process of IR injury and is a dynamic process [25]. Moderate autophagy is beneficial for cell survival against various stress conditions. However, excessive or insufficient autophagy leaves organs vulnerable to IR, so it is necessary to maintain autophagy at a proper level. Currently, contending that upregulated or downregulated autophagy affords protection against organ IR injury is controversial. In hepatic IR injury models, most studies indicate a protective role for autophagy [8, 26], whereas both protective and detrimental roles have been assigned to autophagy in the heart [27] and kidney [28]. These discrepancies might be explained by the fact that autophagy exhibits dual roles in different disease circumstances and is also linked to oxidative stress. Moreover, many studies have showed that administration of autophagy agonists (include RAP) at different time points both before ischemia and at the onset of reperfusion can alleviate IR injury by activating autophagy [29, 30]. In this study, we aimed to confirm the specific protection mechanism of IPO, so the standardization of IPO implementation and operation is very important. In order to reduce the impact of other factors on the implementation of IPO, we have administrated RAP before ischemia. In our current study, autophagy is slightly activated by IIR as a protective strategy, but the degree was not enough to protect the gut. Meanwhile, IPO treatment did sharply increase the expression of LC3 II/I and activate the autophagy to protect the gut against IIR injury. Furthermore, we found that 3-MA significantly inhibited autophagy, enhanced oxidative stress, worsened IIR injury, and abolished the IPO-afforded protection against IIR injury. In contrast, rapamycin notably induced autophagy, attenuated IIR injury, and intensified the protection of IPO. These results revealed that autophagy may be an important component in IPO-afforded protection against IIR injury.

Nrf2, a member of the NF-E2 family of nuclear basic leucine zipper transcription factors, regulates the gene expression of several antioxidative enzymes. Under homeostatic conditions, unactivated Nrf2 is located in the cytoplasm and binds to Keap1, which mediates the rapid ubiquitination and subsequent degradation of Nrf2 by the proteasome. Upon exposure to oxidative stress, Nrf2 is released from the Keap1/Nrf2 complex and translocates to the nucleus to bind to AREs in the promoters of genes encoding antioxidant enzymes, such as HO-1, NQO1, *γ*GCS, and GST [31]. Recent studies have indicated that Nrf2 is in contact with autophagy. NaHS significantly attenuates acrylonitrile-induced oxidative stress by reducing ROS, activating autophagy, and stimulating the nuclear translocation of Nrf2 [32]. Activation of the Nrf2-ARE signaling pathway prevents hyperphosphatemia-induced vascular calcification by inducing autophagy in renal vascular smooth muscle cells [33]. The protective effects of triterpenoid CDDO-imidazole against liver IR injury are attributed to enhanced autophagy, which is mediated by the activation of the Nrf2/HO-1 pathway [34]. In recent years, many studies showed that phosphorylated p62 competitively binds to the KEAP1, activating Nrf2, and creates a positive feedback loop in the p62-Nrf2-KEAP1 pathway [35]. Meanwhile, the autophagic degradation of p62 will break the loop, and the accumulation of small MAF proteins, which occurs upon activation of NRF2, may lead to repression of p62 [36]. These incongruous findings may be caused by differences in the experimental protocols, animal species, and target organs among studies. Our results showed that autophagy proteins, Nrf2, and downstream proteins were increased after IIR insult; meanwhile, our results also showed increased IIR-induced injury, suggesting that the protective effects produced by the increased expression of Nrf2 are still not sufficient to protect tissues fully from IIR-induced injury. Strikingly, IIR-induced gut injury was reversed by IPO and significantly increased the expression of Nrf2 and autophagy-related proteins. Moreover, inhibition of Nrf2 with brusatol caused the accumulation of p62 and reduction of Beclin-1 levels and the LC3 II/I ratio, which point to the interference of autophagy by IPO.

GSK-3*β* is a cytoplasmic serine/threonine protein kinase that regulates numerous cellular processes through a number of signaling pathways important for cell proliferation, stem cell renewal, apoptosis, and development [37]. Some studies have shown that GSK-3*β* is upregulated in many disease states, including neurodegeneration [38], diabetes [39], inflammatory conditions [40], and some cancers [41]. GSK-3*β* is a downstream target of Akt, which serves as a potential modulator of oxidative stress, and is observed to be uniquely dependent on Akt phosphorylation at Ser^473^. The relevance of GSK-3*β* and Nrf2 activation has also been discussed. An increasing body of literature indicates that GSK-3*β* phosphorylates Ser residues in the Neh6 domain of Nrf2 by creating a phosphodegron and promotes Nrf2 degradation in a Keap1-independent manner [42]. In the current study, GSK-3*β* was downregulated by the phosphorylation of Ser^9^, which was accompanied by the upregulation of p-Akt and Nrf2. Conversely, GSK-3*β* expression was significantly recovered with SC66 treatment compared with the control along with decreased Nrf2, which further demonstrated that GSK-3*β* negatively regulated Nrf2. These results indicated that although Keap1 is the major regulator of Nrf2 protein stability, there are other signaling pathways involved in Nrf2 regulation, and the protective effects of IPO are associated with the Akt-mediated inhibition of GSK-3*β* phosphorylation and consequent Nrf2 activation and cytoprotection.

In summary, the present study demonstrated that enhanced autophagy plays a pivotal role in IPO-afforded protection against IIR injury. This phenomenon is due to Nrf2 activation through the Akt/GSK-3*β* pathway. We realized that our study is not without limitations. The signal pathways and regulation mechanisms involved in IIR and IPO are complicated; in this study, we only focus on the relationship of autophagy and GSK-3beta/Nrf2 signaling pathway in the IPO-induced protection. Therefore, further studies are needed to determine the other related mechanisms (such as mPTP, endoplasmic reticulum stress, and relationship with Nrf2 and p62).

## 4. Materials and Methods

### 4.1. Animals

All experiments and procedures were performed in accordance with the *Guide for the Care and Use of Laboratory Animals* by the National Institutes of Health (NIH Publication No. 80-23), Directive 2010/63/EU in Europe, and were approved by the Animal Care Committee of Wuhan University, China. This study was performed in the Animal Center of Renmin Hospital at Wuhan University. Adult male C57BL/6J mice (25 ± 3 g, Hunan Slac JD Laboratory Animal Co., Ltd., Hunan, China) were housed in individual cages in a climate-controlled room (23 ± 1°C; relative humidity 60 ± 5%) with 12 h light-dark cycles with free access to food and water. The mice were acclimated for 2 weeks before the experiments and subjected to a stabilization period after surgery at the Animal Experiment Center of Renmin Hospital at Wuhan University.

### 4.2. Materials and Reagents

Reagent kits to detect D-lactate acid (D-LA), intestinal fatty acid binding protein (I-FABP), diamine oxidase (DAO), malondialdehyde (MDA), superoxide dismutase (SOD), and glutathione/L-glutathione (oxidized) (GSH/GSSG) were obtained from Nanjing Jiancheng Bioengineering Institute (Nanjing, China). A nuclear extract kit was purchased from Beyotime Institute of Biotechnology (Haimen, China). Antibodies to microtubule-associated protein 1 light chain 3 (LC3), Beclin-1, p62, p-Akt (Ser^473^), Akt, p-GSK-3*β* (Ser^9^), GSK-3*β*, glyceraldehyde-3-phosphate dehydrogenase (GAPDH), Nrf2, heme oxygenase 1 (HO-1), NAD(P)H: quinine oxidoreductase 1 (NQO1), and LaminB1 were purchased from Cell Signaling Technology (Danvers, MA, USA). A LI-COR IRDye800CW goat anti-rabbit secondary antibody was obtained from LI-COR Biosciences (Lincoln, NE, USA). CQ was purchased from Cell Signaling Technology, Danvers, MA, USA. The autophagy inducer rapamycin and inhibitor 3-methyladenine (3-MA) were from Selleck Chemicals (Houston, TX, USA). SC66 and brusatol, specific antagonists of Akt and Nrf2, respectively, were purchased from Sigma-Aldrich, Merck KGaA (Darmstadt, Germany). All chemicals used were of the highest grade available.

### 4.3. IIR Model

All animals were fasted for 12 h before the experiments but had free access to water. We used a well-established model of IIR injury [43] in which the mice were anesthetized completely and subjected to IIR by clamping the superior mesenteric artery (SMA) for 45 min followed by reperfusion for 120 min. The SMA was isolated and temporarily occluded with a microvascular clip. The clip was removed gently 45 min later. IPO was implemented before reperfusion, which was performed by 3 cycles of 10 s reperfusion and ischemia at the initiation of reperfusion [19]. Sham-operated mice underwent the same surgical procedures without SMA occlusion. After reperfusion for 120 min, the mice were euthanized. Intestinal tissues and blood were collected and frozen at −70°C for the following experiments.

### 4.4. Experimental Protocol

After surgical preparation, the animals were allocated randomly to the 3 following groups (*n* = 8 in each group): (1) sham (S), (2) IIR, and (3) IPO. Furthermore, to evaluate the effects of autophagy and Akt/GSK-3*β*/Nrf2 on IPO-induced protection, a second set of experiments was performed on the following groups (*n* = 8 in each group): (1) S+vehicle, (2) S+CQ, (3) IIR+CQ, (4) IPO+CQ, (5) S+rapamycin (RAP), (6) IIR+RAP, (7) IPO+RAP, (8) S+3-MA, (9) IIR+3-MA, (10) IPO+3-MA, (11) IPO+vehicle, (12) IPO+SC66, and (13) IPO+brusatol. Mice were pretreated with 0.9% NaCl dissolved in 1% dimethyl sulfoxide (DMSO) as a vehicle; CQ was also dissolved in 1% DMSO and administrated by 50 mg/kg intraperitoneally before experiment; RAP (dissolved in 1% DMSO, 0.25 mg/kg, i.p.) was administered at 1 h before SMA occlusion to induce autophagy [44]; 3-MA (dissolved in 1% DMSO, 30 mg/kg, i.p.) was administered at 1 h prior to ischemia to inhibit autophagy [45]; SC66 (dissolved in 1% DMSO, 25 mg/kg, i.p.) was administered twice a week for 1 week before the experiment to inhibit Akt [46]; and brusatol (dissolved in 1% DMSO, 4 mL/kg, i.p.) was administered once every other day for 10 days before the experiment to inhibit Nrf2 [43].

### 4.5. Histopathology of Intestinal Tissue

Samples were taken from the same part of the small intestines at the distal end of the ileum. All specimens were fixed with 4% paraformaldehyde solution and embedded in paraffin. Subsequently, the paraffin-embedded tissues were cut into 4 *μ*m sections and assessed by hematoxylin and eosin staining under light microscopy. At least 2 different sections from each specimen were examined (original magnification ×200, Olympus BX50; Olympus Optical, Tokyo, Japan). Intestinal mucosal damage was evaluated using improved Chiu's score [47], with blinding to the experimental groups and using a 5-point scale according to the changes of the villi and glands of the intestinal mucosa: grade 0, normal mucosa; grade 1, development of subepithelial Gruenhagen's space at the tip of the villus; grade 2, extension of the space with moderate epithelial lifting; grade 3, massive epithelial lifting with a few denuded villi; grade 4, denuded villi with dilated capillaries; and grade 5, disintegration of the lamina propria, ulceration, and hemorrhage.

### 4.6. Analysis of Intestinal Edema

Tissue edema was detected by the wet/dry (W/D) weight ratio of the gut. At the end of the experiments, 1 cm of small intestine without adipose tissue was taken from the same site in each animal, weighed, and placed in a drying oven at 80°C for 24 h. After drying, the specimens were reweighed, and the ratio of the weight before and after drying was calculated.

### 4.7. Evaluation of Intestinal Barrier Function

D-LA, I-FABP, and DAO in serum were determined using ELISA kits (Nanjing Jiancheng Biocompany, Nanjing, China) in accordance with the manufacturer's instructions.

### 4.8. Evaluation of MDA Level

To determine MDA, tissue (10 mg) was homogenized on ice in 300 *μ*L MDA lysis buffer containing 3 *μ*L BHT (100x). The samples were centrifuged at 13,000 × *g* for 10 min to remove insoluble material. Alternatively, protein was precipitated by homogenizing 10 mg sample in 150 *μ*L water containing 3 *μ*L BHT (100x) and adding 1 volume of 2 N perchloric acid, vortexing, and centrifuging to remove precipitated protein. To achieve this, 200 *μ*L supernatant from each homogenized sample was placed in a microcentrifuge tube. To form MDA-TBA adducts, 600 *μ*L TBA solution was added to each vial containing the standard and sample. The samples were incubated at 95°C for 60 min and cooled quickly to room temperature using an ice bath for 10 min. Then, 200 *μ*L from each reaction mixture, except for the samples, was pipetted into a 96-well plate for analysis. The samples were mixed with 300 *μ*L of 1-butanol and 100 *μ*L of 5 M NaCl with each reaction mixture, vortexed, and centrifuged for 3 min at 16,000 × *g* at room temperature. The 1-butanol layer (top layer) was transferred to a new centrifuge tube, and the 1-butanol was removed either by freeze-drying or by heating on a hot block at 55°C. The remaining material was resuspended in 200 *μ*L ultrapure water. The samples were mixed well, and 200 *μ*L of each sample was added to the wells of a 96-well plate. Fluorescence intensity (*λ*ex = 532/*λ*em = 553 nm) was measured.

### 4.9. Evaluation of SOD Activity

To determine SOD activity, the tissue was rinsed with precooled phosphate-buffered saline (PBS, 0.01 M, pH 7.4) to remove residual blood, and the tissue was cut into small pieces after weighing. The tissue was mixed with a corresponding volume of PBS (generally at a weight/volume ratio of 1 : 9) in a glass homogenizer and ground thoroughly on ice. Finally, the homogenate was centrifuged at 5,000 rpm for 10 min, and the supernatant was taken as the sample to be tested. The samples were mixed with working solution thoroughly, incubated at 37°C for 20 min, and absorbance was read at 450 nm using a microplate reader. SOD activity (inhibition rate %) was calculated using the following equation: {[(Apositive control − Ablank 1) − (Asample − Ablank 2)]/(Apositive control − Ablank 1)} × 100.

### 4.10. Evaluation of GSH/GSSG

To determine the level of reduced and total GSH, the intestines of mice were homogenized in ice-cold 0.01 M HCl and sonicated for 30 s at 4°C. After centrifugation at 14,000 × *g* for 10 min at 4°C, the supernatant was mixed with 5% sulfosalicylic acid (2.5% final concentration) to precipitate the proteins and centrifuged again as described above. For total glutathione (GSH + GSSG), triethanolamine was added to the supernatant to give a final concentration of 6% (vol/vol). For GSSG measurements, 2% (vol/vol; final concentration) of 2-vinylpyridine was also added. The assay buffers contained 1.52 mM NaH_2_PO_4_, 7.6 mM Na_2_HPO_4_, 0.485 mM EDTA, 1 U/mL glutathione reductase, and 0.1 mM NADPH (pH 7.5). After the addition of an aliquot of the sample, the assay mixture was incubated for 2 min. The reaction was started by adding 5,5′-dithiobis-(2-nitrobenzoic acid) to give a final concentration of 0.4 mM. Glutathione concentration was determined spectrophotometrically at a wavelength of 412 nm. GSH content was calculated as the difference between total glutathione and GSSG content.

### 4.11. Transmission Electron Microscopy (TEM)

Intestinal tissue were fixed first in 4% glutaraldehyde and then in 2% osmium tetroxide. After washing with PBS and dehydration in graded ethanol, tissue samples were embedded in epoxy resin. Blocks were cut into ultrathin sections using an ultramicrotome (Ultracut UCT; Leica Microsystems, Germany). Samples were stained with lead citrate. The ultrastructure of tissue sections was observed using a transmission electron microscope (TEM) (H-7000 FA, Hatachi, Japan). Autophagosomes or autolysosomes are characterized by the characteristic structure of a bilayer or multilayer smooth membrane that completely surrounds the compressed mitochondria or membrane-bound electron-dense material.

### 4.12. Nuclear Protein Extraction

Nuclear and cytoplasmic proteins were extracted from frozen intestinal tissues with a nuclear extraction kit (Beyotime Institute of Biotechnology, Haimen, China) according to the manufacturer's instructions [48]. Tissue (60 mg) from each mouse was weighed, cut into small pieces, and homogenized in 200 *μ*L cold lysis buffer. The homogenate was centrifuged for 5 min at 1,500 × *g* at 4°C. The supernatant, which contained the cytoplasmic fraction, was transferred to a fresh tube. To the pellet, 200 *μ*L cell lysis buffer B was added. The sample was incubated on ice for 15 min and centrifuged for 5 min at 12,000 × *g* at 4°C. The supernatant fraction containing cytosolic components was aspirated, and the nuclei were visible as a thin pellet at the bottom of the tube. The supernatant (nuclear fraction) was transferred to a clean microcentrifuge tube, aliquoted, and stored at −80°C until the assay.

### 4.13. Western Blot Analysis

Tissues were weighed, lysed in RIPA buffer (1% NP40, 0.5% sodium deoxycholate, and 0.1% SDS in PBS) and homogenized at 4°C using a TissueRuptor (QIAGEN, Hilden, Germany). The total protein concentration of the supernatant was determined by a BCA assay. Samples were loaded on 12% (for LC3, Beclin-1, p62, p-Akt (Ser^473^), Akt, p-GSK-3*β* (Ser^9^), GSK-3*β*, HO-1, NQO1, and GAPDH) or 8% (for Nrf2 and LaminB1) SDS-PAGE at 100 V for 90 min. After electrophoresis, the proteins were transferred onto PVDF membranes (Thermo Fisher Scientific, Inc. Waltham, MA, USA) at 200 mA for 90 min. The membranes were blocked for 2 h with 5% BSA (Bio-Rad Laboratories, Hercules, CA) in TBS (10 mM Tris, 100 mM NaCl) and incubated overnight at 4°C with rabbit anti-mouse antibodies to LC3 (1 : 500), Beclin-1 (1 : 400), p62 (1 : 1,000), Nrf2 (1 : 200), HO-1 (1 : 200), NQO1 (1 : 1,000), Akt (1 : 1,000), p-Akt (Ser^473^) (1 : 1,000), GSK-3*β* (1 : 1,000), p-GSK-3*β* (Ser^9^) (1 : 1,000), GAPDH (1 : 1,000), and LaminB1 (1 : 200) (all from Cell Signaling Technology, Danvers, MA, USA). After washing three times with TBS with Tween 20, the membranes were incubated with the corresponding LI-COR IRDye800CW goat anti-rabbit secondary antibody (1 : 10,000; LI-COR Biosciences, Lincoln, NE, USA) for 1 h at room temperature. The intensity of the identified bands was detected using the Odyssey two-color infrared laser imaging system, and densitometry was carried out using Odyssey software (both from LI-COR Biosciences) [11].

### 4.14. Statistical Analysis

Data are expressed as the mean ± SD. GraphPad Prism 6.0 (GraphPad Software, Inc., San Diego, CA, USA) was used to manage the data and calculate the results. Statistical evaluation of the data was performed by one-way analysis of variance followed by Tukey's post hoc test; *p* < 0.05 was considered statistically significant.

## Data Availability

The authors declare that all the data and materials supporting the findings of this study are available upon reasonable request.

## Abbreviations

IPO: Ischemic postconditioning
IR: Ischemia-reperfusion
IIR: Intestinal ischemia-reperfusion
SIRS: Systemic inflammatory response syndrome
MODS: Multiple organ dysfunction syndrome
ROS: Reactive oxygen species
IPC: Ischemic preconditioning
S: Sham
W/D: Wet/dry weight
DAO: Diamine oxidase
D-LA: D-Lactate acid
I-FABP: Intestinal fatty acid binding protein
MDA: Malondialdehyde
SOD: Superoxide dismutase
GSH: Glutathione
GSSG: L-Glutathione (oxidized)
ELISA: Enzyme-linked immunosorbent assay
LC3 II/I: Microtubule-associated protein 1 light chain 3 II/I
3-MA: 3-Methyladenine
RAP: Rapamycin
SMA: Superior mesenteric artery
PBS: Phosphate-buffered saline
GSK-3*β*: Glycogen synthase kinase 3 beta
Nrf2: Nuclear factor (erythroid-derived 2)-like 2
ARE: Antioxidant responsive element
HO-1: Heme oxygenase 1
GAPDH: Glyceraldehyde-3-phosphate dehydrogenase
NQO1: NAD(P)H: quinine oxidoreductase 1
DMSO: Dimethyl sulfoxide
SD: Standard deviation
mPTP: Mitochondrial permeability transition pore
TEM: Transmission electron microscopy
SDS: Sodium dodecyl sulfate
TBS: Tris-buffered saline.
